# Effect of the Alkali Pretreatment on the Structure and Properties of Bamboo-Based Porous Molding Materials

**DOI:** 10.3390/polym17233166

**Published:** 2025-11-28

**Authors:** Baoyong Liu, Weichen Li, Xiaowei Zhuang, Xin Pan, Hui Qiao, Yongshun Feng

**Affiliations:** 1 College of Environmental Science and Engineering, Liaoning Technical University, Zhonghua Road 47, Fuxin 125105, China; liubaoyong@lntu.edu.cn (B.L.); 472321912@stu.lntu.edu.cn (W.L.); 2 Institute of Chemical Processing of Forest Products, Zhejiang Academy of Forestry, Liuhe Road 399, Hangzhou 310023, China; zhuangxiaowei@zjforestry.ac.cn (X.Z.); panxin@zjforestry.ac.cn (X.P.); qiaohui@zjforestry.ac.cn (H.Q.)

**Keywords:** bamboo cellulose, suspension, biodegradable, molding material, alkali pretreatment

## Abstract

The development of novel materials from biomass is a potential alternative to replace traditional petrochemical resources. In accordance with the “Bamboo Substitute Plastic” initiative, bamboo-based lightweight porous materials are a class of foam materials fully prepared from biomass resources with a lightweight and high-strength structure. However, issues such as excessive lignin content and uneven pore structure distribution within these materials hinder their application. This study utilized bamboo powder as a raw material to prepare lightweight, porous molding materials through a hydrothermal grinding process. The influence of different concentrations of alkaline pretreatment was investigated. The fabricated molding material had a density of 0.36–0.49 g/cm^3^ at 80 °C and 0.32–0.38 g/cm^3^ at 105 °C. Samples dried at 80 °C had a water absorption of 161% to 304%, while those dried at 105 °C had a water absorption of 223% to 305%. The wet swelling was characterized by volume expansion from 6.2% to 7.7%. The surface of the molding materials became increasingly homogeneous without any cracks due to the alkali pretreatment. FTIR data showed that more surface hydroxyl groups were observed after alkaline pretreatment, and some carbonyl groups in the hemicellulose structure were removed; meanwhile, the crystallinity index after alkaline pretreatment was higher than that of untreated bamboo. The alkali solution was proposed to remove part of the lignin and improve the fibrillation degree of the bamboo fibers. The highest tensile strength of the samples was 9.63 MPa, while the highest compressive strength obtained was 0.92 MPa under the alkali treatment. With lightweight and fully degradable properties, the bamboo-based porous molding materials have promising application prospects in environmental protection, construction, packaging, and related fields.

## 1. Introduction

Replacing non-renewable fossil resources with sustainable biomass and utilizing the versatility of biomass are crucial for achieving economic and environmental development at this stage [1]. Unlocking the conversion of cellulose, hemicellulose, and lignin through selective condensation into active compounds and producing new chemicals via condensation-driven processes is a novel approach to achieving the efficient utilization of biomass [2]. In recent years, numerous research teams have developed lightweight porous materials characterized by low density, high porosity, and large specific surface area [3,4,5]. Through the combination of microfluidic processing and freeze-drying, wood nanocellulose could be fabricated into ultra-lightweight foams with a porosity of 89% and a thermal conductivity as low as 34 mW/m·K, whose performance was comparable to that of traditional polystyrene foams [6]. With advancements in rotational molding technology, wood flour/polyethylene composite foams could now be produced industrially, achieving a flexural modulus of up to 655 MPa while maintaining a low density of 0.45–0.72 g/cm^3^ [7]. Additionally, the high-value utilization of wood waste has emerged as a new research focus. Heat-insulating foams with controllable pore structures could be prepared from pine beetle-killed wood following alkali treatment, and mechanical foaming of a tannin-lignin composite system yields self-supporting porous materials with a compressive modulus of 22.9 MPa [8].

As a representative of bio-based lightweight porous materials, wood foam has demonstrated unique advantages in lightweight thermal insulation, sound absorption, and buffering applications [9]. Wood foam was initially developed by Germany’s Fraunhofer Institute for Wood Research as a category of lightweight foam materials with wood fibers as their core structure [10]. It can be biodegraded by microorganisms in the natural environment, thereby reducing environmental pollution. In cold chain transportation, such foam materials not only achieve thermal insulation through their honeycomb structure but can also be directly integrated into paper recycling processes after use to meet circular economy requirements. In construction industry applications, the open-cell structure formed by internally intertwined wood fibers endows wood foam with excellent sound absorption properties. Additionally, it can serve as a sandwich layer to replace traditional polystyrene boards. Moreover, oriented strand board (OSB) produced via hot pressing processes combines compressive strength with lightweight properties, enabling it to replace polyurethane fillers in furniture manufacturing [11].

Compared with traditional wood, bamboo’s superior properties [12] and unique porous structure endow it with greater process ability [13]; its rapid growth rate and high strength have gradually established it as a biomass resource capable of replacing fossil fuels [14,15], while also demonstrating immense potential in the field of lightweight porous materials [13]. Bamboo resources are widely distributed globally and play a significant role in the green economy, sustainable development, and addressing global climate change [16]. In 2022, the “Bamboo as a Substitute for Plastic” initiative was elevated to an international cooperation agenda, with China and the International Bamboo and Rattan Organization (INBAR) jointly advancing global research, development, and application of bamboo-based materials. However, bamboo is susceptible to cracking and deformation [17] and exhibits hygroscopic expansion as moisture content fluctuates [18]. A series of issues, including excessively high lignin content and uneven distribution of internal pore structures, has impeded its broader applications. Current research focuses on using green modification technologies to optimize the pore structure of bamboo, enhance interface stability and intrinsic bonding strength, and promote the rational utilization of waste resources.

For the porous, lightweight materials, bamboo exhibited inferior properties, including shrinkage and cracking, compared to pine and beech materials [19]. A pretreatment step is necessary for the following fabrication process. Alkali treatment is one of the most widely used and cost-effective methods [13]. Numerous studies have demonstrated that alkali treatment has a significant influence on the structure and properties of natural fibers, such as wood. Alkali pretreatment plays a crucial role in determining the structural properties of bamboo [13]. Additionally, bamboo fibers exhibit enhanced flexural and compressive strength after undergoing alkali treatment [20]. Alkali pretreatment can dissolve lignin and hemicellulose, disrupt the chemical bonds between them, and reduce the crystallinity and degree of polymerization of cellulose. During alkali pretreatment, the ester bonds between lignin and carbohydrate polymers are broken, which can substantially enhance the bonding strength of the internal fibers within bamboo itself [15,21,22,23,24]. Although existing studies have revealed the regulatory effect of alkali pretreatment on the microstructure of bamboo fibers, there are still significant limitations: first, most studies focused on composite materials, with fewer studies on alkali treatment of bio-based materials; second, most studies investigated the effects on bamboo fibers from the perspectives of temperature and alkali treatment, or directly from the concentration of alkali treatment, while few combined preparation technology with alkali treatment; third, there is a lack of research on the resource utilization of bamboo waste to make lightweight molding materials, making it challenging to meet the demands of green production [25].

Based on traditional delignification processes, this study introduces high-energy hydrothermal grinding technology to systematically investigate bamboo-based lightweight porous materials, focusing on the following aspects: the effectiveness of NaOH pretreatment in removing lignin from bamboo powder; its effects on the surface morphology, hygroscopicity, functional groups, and microstructure of the materials; and its influence on pore structure and mechanical properties. Although bamboo-based porous molded materials show significant potential as eco-friendly alternatives to petroleum-based foams, systematic studies remain insufficient regarding how alkaline pretreatment concentration regulates lignin removal efficiency, fiber fibrillation, and the resulting structure–property relationships, such as pore uniformity, mechanical strength, and water absorption. We hypothesize that pretreatment at an optimal alkali concentration can selectively remove partial lignin, enhance fiber fibrillation, and thereby homogenize the pore structure of the resulting porous materials. This structural optimization is expected to improve mechanical properties while maintaining favorable lightweight and water absorption characteristics, ultimately overcoming key bottlenecks that limit their practical application.

## 2. Materials and Methods

### 2.1. Materials

Bamboo powder was purchased from Zhumeng Bamboo Technology Company (Xuancheng, China). The bamboo powder was screened through a 20-mesh (0.85 mm) sieve to remove impurities and obtain a uniform particle size. Sodium hydroxide (NaOH, analytical grade) was procured from Shanghai Lingfeng Chemical Reagent Company (Shanghai, China).

### 2.2. Fabrication of the Bamboo-Based Molding Materials

**Figure 1:**
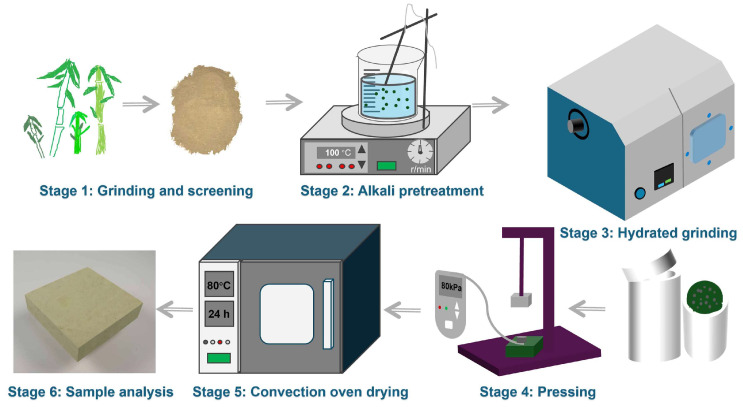
The fabrication procedure of bamboo-based porous molding materials.

NaOH solution was prepared at a concentration of 0.5 wt.%, 1.0 wt.%, 1.5 wt.%, 2.0 wt.%, and 2.5 wt.%. The blank one was labeled as 0.0 wt.%. A weight of 50 g bamboo powder was added to 1 L NaOH solution in a 2 L flask. It was heated to the boiling point with a stir rate of 600 rpm for one hour. After cooling, it was washed in a polyethylene mesh bag with distilled water until the pH reached neutrality and then dried at 105 °C to remove any remaining water. A weight of 30 g pretreated bamboo powder was mixed with 150 g distilled water at a ratio of 1:5 and placed into a 500 mL zirconia grinding pot (Model 3SP2, Nanda Instrument, Nanjing, China). The hydrothermal grinding time was set for 2 h. The resulting suspension was transferred into a high-pressure reactor (Model 4547, Parr Instrument, Moline, IL, USA) for further refinery. The reaction time was 30 min, and the temperature was 120 °C, with a pressure of 0.6 MPa and a rotating speed of 600 rpm. The refined bamboo fiber suspension was collected and placed in a 7 cm × 7 cm × 5 cm mold. A pressure of 80 kPa was applied to the suspension for 20 min to remove part of the water and form a fixed shape. A fibrous mat was obtained after the removal of the mold. It was further dried in an oven at 80 °C and 105 °C, respectively. During drying, the mat was weighed every hour to monitor the moisture loss. The surface of the dried products was removed for a better observation of fiber morphology. The fabrication procedure is presented in Figure 1.

### 2.3. FTIR Analysis of the Bamboo-Based Porous Molding Materials

The functional groups of bamboo-based porous molding materials were analyzed by Fourier Transform Infrared Spectroscopy (Nicolet iS 5, Thermo Fisher Scientific, Waltham, MA, USA). The samples were dried at 103 °C to remove the free water. The KBr pressed-pellet technique was used to make the tablet. The samples were dried and ground into powder, then mixed with KBr to prepare the transparent pellets. Spectral acquisition was performed in the range of 650–4000 cm^−1^ from 32 scans with a resolution of 2 cm^−1^. The baseline was calibrated prior to each test.

### 2.4. XRD Analysis of the Bamboo-Based Porous Molding Materials

The crystal structures of the bamboo raw material and bamboo suspension were determined by X-ray diffraction (Rigaku SmartLab, Akishima, Japan) using Cu Kα radiation with a wavelength λ = 0.154 nm. The measurements were conducted over a 2θ range of 5–40° at a scanning rate of 4°/min, with a step size of 0.02°, a tube voltage of 40 kV, and a tube current of 30 mA. The crystallinity index of (CrI) the bamboo fibers was calculated using the Segal empirical method:

$$CrI=\frac{I_{002}-I_{am}}{I_{002}}\times 100$$

In the formula, CrI is the crystallinity index (%), I_002_ is the maximum index of the crystallinity peak, and I_am_ is the minimum intensity between the two peaks.

### 2.5. Tests of the Mechanial Performance of the Molding Materials

Mechanical performance was tested using a texture analyzer (SMS-TA Plus-C, Stable Micro Systems, Godalming, UK). Tensile strength and compressive strength were used to evaluate the mechanical strength. The tensile strength was tested using a three-point bend rig in compression mode. The pre-test speed and test speed were set at 0.5 mm/s, while the post-test speed was set at 1.0 mm/s. The compressive strength was tested using a cylindrical probe in the mode of compression. The pre-test speed and test speed were set at 1.0 mm/s, while the post-test speed was set at 10.0 mm/s. The target mode was set at a 5 mm distance with a trigger force of 50 g. The data acquisition rate was 200 pulses per second (pps). All tests were performed in triplicate, and the average values were taken for analysis.

## 3. Results and Discussion

### 3.1. Morphologies of Bamboo-Based Porous Molding Materials

**Figure 2:**
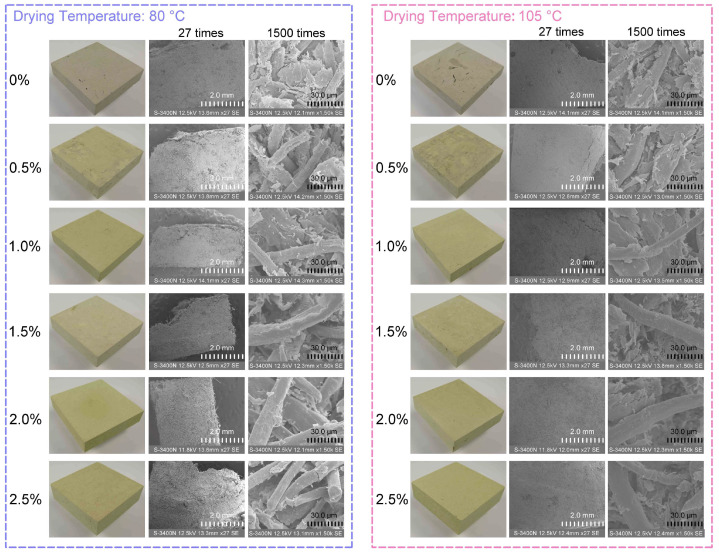
SEM images of bamboo cellulose pretreated with different alkali concentrations.

The images of bamboo cellulose-based molding materials and the fiber morphologies are presented in Figure 2. Open pores of various sizes were found in all the samples, indicating an open-pore structure in the molding materials. The samples without alkali pretreatment had several cracks that could be found on the surface. The surface became smooth, and the cracks disappeared after alkali pretreatment. As the concentration of alkali pretreatment increased from 0.5% to 2.5%, the surface of the molding materials became increasingly homogeneous. The lignin, hemicellulose, wax, and other impurities on the fiber surface were removed through dilute alkali pretreatment. The SEM images show that fibrillation occurs with an increase in NaOH concentration, resulting in increased smoothness of the bamboo fibers. This process increases the spacing between cellulose molecular chains and weakens the strength of hydrogen bonds, leading to cell wall thickening, cavity closure, disruption of the middle layer, fiber separation, and twisting, which ultimately result in more significant changes in surface roughness [24]. The released long fibers enable more effective enhancement of adhesion values through mechanical interlocking [26]. It has been reported that alkali treatment induces the expansion of the cellulose lattice and the hydrolysis of hemicellulose, thereby disrupting the tight arrangement of cell walls and exposing fibers, which enhances their entanglement [27]. Additionally, it enhances delignification efficiency, activates uncondensed lignin, partially degrades hemicellulose, and slightly degrades cellulose, thereby improving its adhesiveness [16]. Through saponification, NaOH solution reacts with ether bonds, ester bonds, and other linkages in the aromatic and aliphatic structures of lignin, selectively breaking hydrogen bonds between cellulose components and reducing crystallinity and degree of polymerization to facilitate more efficient bioconversion [28]. After the hydrothermal grinding process, a fiber matrix is formed with nano, micro, and macro cellulose fibers. After a sequential preparation procedure, the irregularly arranged structure of the molding material is reorganized, resulting in enhanced bonding strength and mechanical performance.

### 3.2. Moisture Loss of the Wet Fibrous Mat During the Drying Process

**Figure 3:**
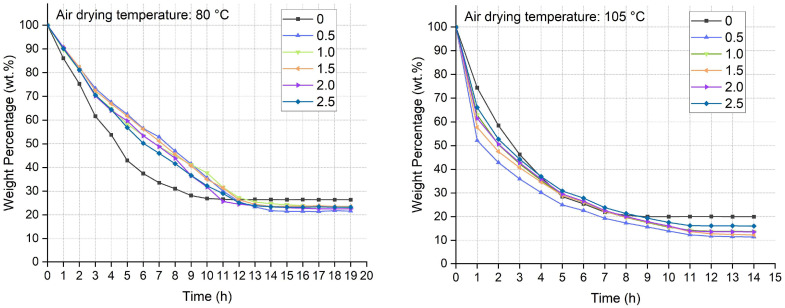
Weight loss of the bamboo fibrous wet mat during oven drying.

The high-viscosity fibrous suspension produced by the HTG process contained around 83 wt.% water. An amount of ca. 47 wt.% water in the suspension was removed by cold pressing at 80 kPa for 20 min [29]. The rest of the water was removed by high-temperature oven drying at 80 °C and 105 °C, respectively. The elimination of water required 12 h at 80 °C and 10 h at 105 °C, as shown in Figure 3. This reflects the dynamic changes in heat transfer and moisture migration throughout the drying process. The concentration of NaOH solution had a very slight influence on the drying rate. At both temperatures, the weight loss trends were similar, with a maximum weight loss occurring in the initial stages. At the first hour, the temperature of the drying medium increased rapidly with an increase in the surface of the samples, the moisture in the mat could absorb more energy per unit time [30]. The suspension is a matrix composed of nano-, micro-, and macro-bamboo fibers. During the drying process, the transfer of moisture within the mat occurs through both capillary action and diffusion. Other than the water removal, the high temperature caused the polymerization of uncondensed lignin and other active components. In this process, water improves the plasticity of the bamboo fibers and gives the final products mechanical strength. For such fully biomass foams, water was even used to weld foams into various designed structures via oven drying [31].

Moisture has a significant impact on the dimensional stability of porous molding materials. Water molecules can saturate the hydroxyl groups in cellulose cell walls, and during drying, the bond energy between cellulose and non-cellulosic components is relatively low [32]. Although water is removed by oven drying, elevated temperatures may induce morphological changes in the samples. This is because excessive drying temperatures or overly rapid drying rates cause internal moisture in the bamboo to diffuse outward rapidly [33]. Therefore, temperature is a critical factor influencing both drying efficiency and sample quality. An appropriate drying temperature or drying procedure must be set to ensure the formed bamboo-based lightweight porous material is free from defects.

### 3.3. Density and Swelling Property of Bamboo-Based Porous Molding Materials

**Figure 4:**
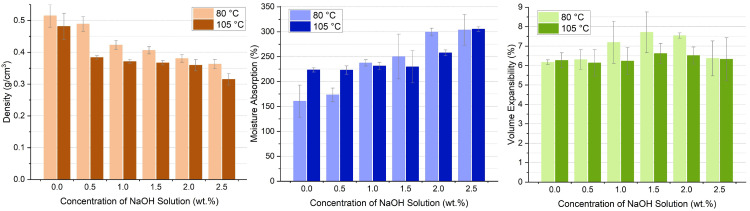
Density and water absorption properties of bamboo-based porous molding materials.

The bamboo-based porous molding material is fabricated without the use of any petrochemical adhesives. The density of molding material was 0.51 g/cm^3^ at 80 °C and 0.48 g/cm^3^ at 105 °C, shown in Figure 4. The samples that were dried at 105 °C had a lower density than those dried at 80 °C. After alkali treatment, the density ranged from 0.36 to 0.49 g/cm^3^ at 80 °C and 0.32–0.38 g/cm^3^ at 105 °C. As the NaOH concentration increased, the density gradually decreased in a regular trend. At the same temperature, samples with higher NaOH concentrations exhibited lower densities, likely due to the increased porosity and reduced moisture content. The minimum density was achieved at the NaOH concentration of 2.5 wt.%. Lignin was partially removed from the bamboo structure by alkali pretreatment. Bamboo fibers of various sizes were generated by the HTG process. These fibers wrap around each other to form a structure that is similar to a random coil. More open pores were formed due to this structure, which made the density of alkali-treated samples lower than that of the untreated samples. However, under high-temperature treatment, cellulose chains break, causing pores to become blocked and thereby reducing porosity. A reduction in porosity enhances sample strength, with the extent of this effect depending on internal structural characteristics [34]. Additionally, higher temperatures intensify molecular motion and result in a more compact structure, which explains why the density of samples treated at 105 °C is lower than that of those treated at 80 °C.

As a porous material derived from lignocellulosic resources, bamboo-based molding products possess high water absorption properties. Samples dried at 80 °C had a water absorption of 161% to 304%, while those dried at 105 °C had a water absorption of 223% to 305%. Water absorption is closely related to the density of samples, shown in Figure 3. The samples with lower density had higher water absorption ability due to more pores existing in the structure. Another factor influencing water absorption is the amount of hydrophilic chemical groups. Both cellulose and hemicellulose in bamboo contain free hydroxyl groups, which readily form hydrogen bonds with water. More free hydroxyl groups are exposed by alkali treatment. Furthermore, Alkali treatment increases the irregularity and roughness of fiber surfaces, which in turn improves the percentage of water absorption [35]. Water absorption can induce dimensional changes in samples, resulting in hygroscopic swelling. This hygroscopic swelling causes variations in the size and volume of wood. In the NaOH concentration range of 0–1.5 wt.%, volume expansion increased from 6.2% to 7.7% at 80 °C and from 6.3% to 6.6% at 105 °C. The phenomenon of water absorption and wet swelling reflects the conversion between hydrogen bonds and non-hydrogen bonds [34]. After high-concentration treatment, fluctuations occur as cellulose rearranges and packs tightly, leading to reduced wet swelling.

### 3.4. FTIR and XRD of Bamboo-Based Porous Molding Materials

**Figure 5:**
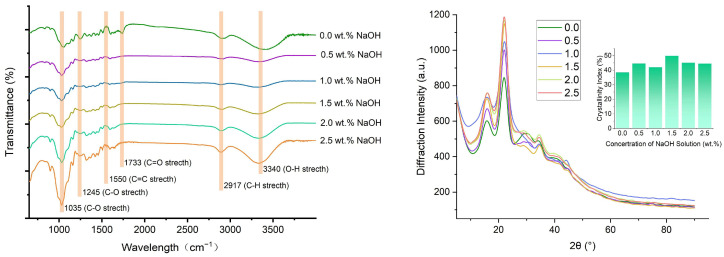
FTIR spectra and X-ray diffraction of bamboo fiber matrix.

The chemical structure of bamboo powder was characterized using FTIR, and the changes in functional groups between untreated and alkali-pretreated samples were analyzed within the wavenumber range of 650–4000 cm^−1^, as shown in Figure 5. The peak at 3340 cm^−1^ corresponds to O–H stretching vibrations, which are associated with intermolecular hydrogen bonds and free hydroxyl groups primarily derived from cellulose, hemicellulose, and lignin [36]. The peak at 2917 cm^−1^ corresponds to C–H stretching vibrations. The changes in these two peaks indicate that more surface hydroxyl groups were observed after alkali pretreatment. The peak at 1733 cm^−1^ which represents the stretching vibration of the C=O groups disappeared after alkali pretreatment. It is often attributed to free carboxyl groups and acetyl carboxylic acids in hemicellulose. Part of the carbonyl groups in the hemicellulose structure were removed after NaOH pretreatment. Peaks at 1550 cm^−1^ represent the C=C stretching vibration in the lignin units, while peaks at 1245 cm^−1^ and 1035 cm^−1^ represent the C–O stretching vibration in the lignin units. The changes in peaks at these areas indicated that the dilute alkali solution pretreatment effectively altered the lignin structure. Combined with the HTG process, the degraded lignin was dissolved in water to yield a soluble fraction consisting of guaiacyl and syringyl monomers and oligomers. The uncondensed lignin with high reactivity was formed. The resulting uncondensed lignin and phenolic compounds contributed to the mechanical performance of the samplers through the polymerization in the subsequent high-temperature drying process [37].

The XRD patterns of the NaOH-pretreated and untreated bamboo samples are shown in Figure 5. Crystallinity influences mechanical properties, density, permeability, and other characteristics of the molding products. The distinct characteristic peaks at 16.5° and 22.1° correspond to the typical cellulose I structure [38]. These peaks are found in all samples, indicating the retention of cellulose I structure after alkali pretreatment [24]. However, the crystallinity index of the untreated bamboo was 38.5%, while that of the pretreated samples ranged from 42.0% to 50.0%. The increase in the crystallinity index indicates that the pretreatment of NaOH solution removes the amorphous substances (lignin, pectin, and hemicellulose) from the cellulose and retains the primary crystalline structure [27]. Higher crystallinity also implies the formation of microfibrils during the HTG process. The improvement of crystallinity means that the arrangement of cellulose molecular chains is more orderly and the intermolecular forces are stronger, which directly endows the fibers with better mechanical strength, thermal stability, and chemical stability. The fiber defibration and lignin reconstitution impart a self-adhering property to bamboo [39]. Additionally, the cellulose in bamboo fiber bundles exhibits an increasing trend from the inside out. In contrast, the angle and crystallinity of cellulose microfibrils show regular fluctuations along the radial direction of the bamboo culm [40]. Furthermore, the rearrangement of crystalline regions increases crystallinity, resulting in more pronounced crystalline properties [24].

**Figure 6:**
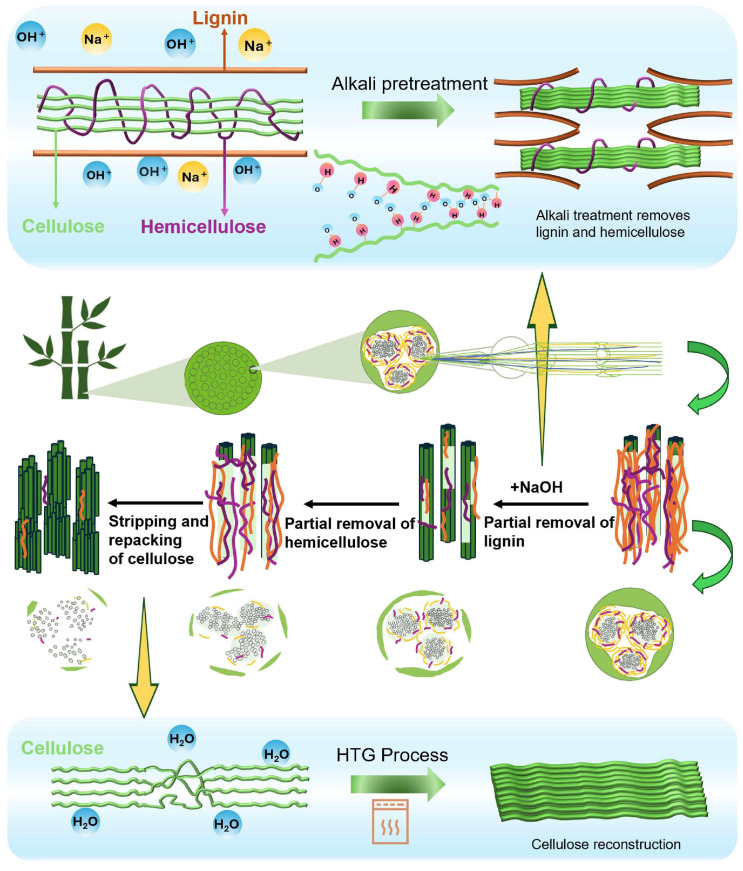
The mechanism of alkali pretreatment combined with the HTG process.

As an efficient bamboo fiber modification technology, alkali treatment fundamentally optimizes the microstructural and macroscopic properties of fibers by precisely removing lignin and hemicellulose from bamboo cell walls in a staged process. In the initial stage of alkali treatment, the NaOH solution triggers the cleavage of ether bonds and carbon-carbon bonds inside lignin molecules. The cellulose microfibrils, originally bound, are gradually released. Subsequently, the residual hemicellulose in bamboo cell walls, surface wax, pectin, and other impurities are effectively removed. The removal of these impurities not only eliminates the physical wrapping of hemicellulose on cellulose but also significantly increases the specific surface area of the fibers with a large number of hydroxyl groups on the surface. A possible detailed mechanism is shown in Figure 6. As active groups, these hydroxyl groups can form stronger chemical bonding and physical adsorption with matrix materials, thereby effectively enhancing the interfacial bonding ability between fibers and the matrix. When bamboo treated with alkali undergoes the HTG process, the fiber and matrix with a gradient structure and heterogeneity undergo more complex structural reorganization.

### 3.5. Mechanical Strength of Bamboo-Based Porous Molding Materials

**Figure 7:**
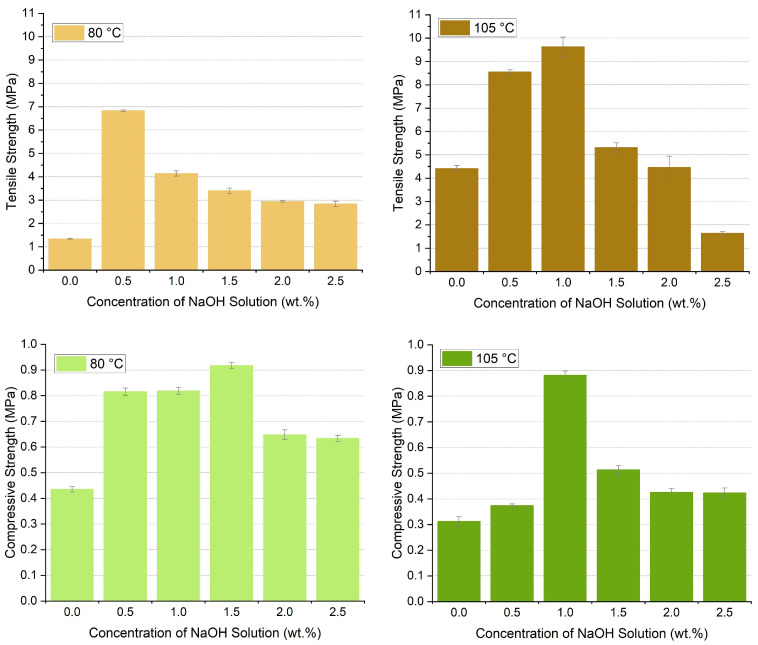
Tensile strength and compressive strength of bamboo-based porous molding materials.

The tensile strength and compressive strength are used to evaluate the mechanical strength of bamboo-based molding materials. The results indicate that alkali pretreatment has a positive effect on improving the mechanical strength of molding materials, as shown in Figure 7. For the tensile strength at 80 °C, it increased from 1.34 MPa to 6.83 MPa with 0.5 wt.% alkali pretreatment but decreased to 2.84 MPa as the NaOH concentration increased to 2.5 wt.%. A similar phenomenon was observed for samples dried at 105 °C, with the highest tensile strength of 9.63 MPa. The tensile strength of bamboo fiber is closely related to alkali treatment conditions: when NaOH concentration exceeds 0.5%, excessive lignin removal and cellulose degradation lead to fiber strength reduction. High-temperature drying (105 °C) might promote water gasification and pore densification. Stronger alkali treatments negatively impacted fiber which was verified by other research [13]. For the compressive strength, it increased with the NaOH concentration but decreased above the NaOH concentration of 2.0%. The highest compressive strength was obtained with 0.92 MPa with 0.5 wt.% NaOH concentration at 80 °C. Compression strength is regulated by pore structure: 1.0% NaOH concentration balances pore uniformity and fiber reinforcement effects. The differences in tensile and compressive performance originate from distinct dependency mechanisms on fiber integrity and matrix structure.

During the HTG process with alkali-pretreated bamboo powder, the residual cellulose microfibrils spontaneously rearrange under the combined action of intermolecular hydrogen bonds and van der Waals forces, gradually forming a cellulose crystalline structure with a regular structure and tight arrangement. The formation of this structure further optimizes the internal structure of the fibers, providing structural support for the comprehensive improvement of their properties. It is worth noting that the fiber defibrillation and lignin reconstruction that occur during alkali treatment endow bamboo with unique self-adhesion properties, and the formation of this property is closely related to structural heterogeneity. In the absence of any adhesives, mechanical strength relies on the bamboo’s inherent bonding properties. The mechanical strength is attributed to two mechanisms. The first is to activate wood’s own binding ingredients, and the second is the anchorage of cellulose fibers [10]. The two mechanisms can be achieved through the HTG process described in a previous study [29]. The alkali pretreatment enhances the HTG process by activating the internal bonding forces within the bamboo. The strength of the samples stems from the internal bonding capacity of bamboo fibers, which is determined by the contact surfaces of cross fibers and fiber fragments and is independent of fiber length. The anatomical structure of the fibers also affects the mechanical strength and properties of bamboo [32]. Due to the alkali pretreatment, longer bamboo fibers, as observed in the SEM images, result in a higher anchorage degree. Nevertheless, excessively high concentrations damage the cellulose fibers themselves, resulting in a certain degree of fiber loss and affecting intermolecular forces, which leads to reduced strength [41]. A high mechanical strength of bamboo-based molding material can be achieved by a careful design of alkali pretreatment.

## 4. Conclusions

With alkali pretreatment, a bamboo-based, porous, lightweight material was obtained through a hydrothermal grinding process. A high-viscosity bamboo fiber suspension with nano-, micro-, and macro-scale features was obtained, with a water content of approximately 83 wt.%. The elimination of water required 12 h at 80 °C and 10 h at 105 °C by oven drying. The surface properties of the products were significantly improved by diluting the NaOH solutions. The smoothness of the bamboo fibers increased, and the crystallinity of bamboo fibers was enhanced by alkali pretreatment. The released long fibers enable more effective enhancement of adhesion values through fiber anchorage The molding products had a density in the range of 0.32–0.49 g/cm^3^. It was an open core structure with bamboo fibers wrapped around each other. The products dried at 80 °C had a water absorption of 161% to 304% while at 105 °C had a water absorption of 223% to 305%. The volume expansion was lower than 8% after immersion in cold water for 24 h. Stronger alkali treatments might influence the mechanical performance of the bamboo-based molding materials negatively. The present study offers a viable pathway for the low-carbon utilization of bamboo through the rational exploitation of biomass resources. However, the research remains at the laboratory stage, and key process parameters such as drying temperature require systematic optimization to better regulate pore structure and mechanical properties. Moreover, current evaluations primarily focus on short-term water absorption and swelling behavior, lacking long-term durability assessments under cyclic hygrothermal conditions or complex natural environments—such as thermal aging, UV aging, and biodegradation. Future work should prioritize process optimization and pilot-scale trials, conduct long-term performance evaluations, and enhance material durability through surface coating or cross-linking modifications.

## Data Availability

The original contributions presented in this study are included in the article. Further inquiries can be directed to the corresponding authors.

## Abbreviations

The following abbreviation is used in this manuscript:

HTG	Hydrothermal Grinding
